# Dai-Huang-Fu-Zi-Tang alleviates pulmonary and intestinal injury with severe acute pancreatitis via regulating aquaporins in rats

**DOI:** 10.1186/s12906-017-1789-x

**Published:** 2017-06-02

**Authors:** Xin Kang, Xiao-Guang Lu, Li-Bin Zhan, Zheng-Kai Liang, Wen-Xiu Guo, Qi Ma, Yi Wang, Jian-Bo Song, Jin-Yu Feng, Cong-Han Wang, Li-Zhi Bai, Yi Song, Guo-Hui Liu

**Affiliations:** 1 Department of Emergency Medicine, Zhongshan Hospital, Dalian University, Dalian, 116001 China; 2 Basic Medical College, Nanjing University of Chinese medicine, Nanjing, 210023 China; 3 Department of Surgery, Liaocheng People’s Hospital, Shandong, 300100 China; 4 Graduate School, Dalian Medical University, Dalian, 116044 China; 5 Department of Emergency Medicine, First Hospital of Jilin University, Changchun, 130012 China

**Keywords:** Severe acute pancreatitis, Dai-Huang-Fu-Zi-Tang, Aquaporin, Injury

## Abstract

**Background:**

Dai-Huang-Fu-Zi-Tang (DHFZT) is a famous traditional Chinese prescription with intestinal obstruction, acute pancreatitis and cholecystalgia for thousands of years. Our previous work found that DHFZT could act against pulmonary and intestinal pathological injury in rats with severe acute pancreatitis (SAP). But the underlying mechanism has not been fully elucidated. The aim of present study was to investigate whether DHFZT could relieve pulmonary and intestinal injury by regulating aquaporins after SAP induced by sodium taurocholate in rats.

**Methods:**

Forty of SD rats were used for dose dependant experiments of DHFZT.Accurate-mass Time-of-flight liquid chromatography-mass spectrometry was used for qualitative screening of chemical compositions of DHFZT. Twenty-four rats were randomly divided into 3 groups: sham group (*n* = 8), model group (SAP, *n* = 8), DHFZT group (SAP with DHFZT treatment, *n* = 8). SAP models were established by retrograde injections of 5% sodium taurocholate solutions into rat pancreaticobiliary ducts. Blood samples were taken at 0, 12, 24, 48 h post-operation for detecting serum amylase, lipase, endotoxin, TNF-α, IL-6 and IL-10. Protein expression and location of aquaporin (AQP)1, 5, 8 and 9 were assessed by immunohistochemistry, western blot and immunofluorescence respectively.

**Results:**

The study showed that 27 kinds of chemical composition were identified, including 10 kinds in positive ion mode and 17 kinds in negative ion mode. The results showed that AQP1, AQP5 of lung, and AQP1, AQP5, AQP8 of intestine in model group were significantly lower than that of sham group (*P* < 0.05), and which were obviously reversed by treatment with DHFZT. In addition, protein levels of pro-inflammatory cytokines such as TNF-α, IL-6 and endotoxin in peripheral blood were significantly suppressed by DHFZT, and that anti-inflammatory cytokine like IL-10 was just opposite. Finally, we also noted that DHFZT reduced serum levels of amylase, lipase and endotoxin, and also improved edema and pathological scores of lung and intestine after SAP.

**Conclusions:**

DHFZT ameliorated the pulmonary and intestinal edema and injury induced by SAP via the upregulation of different AQPs in lung and intestine, and suppressed TNF-α, IL-6 expression and enhanced IL-10 expression.

## Background

Acute pancreatitis (AP) is an acute inflammatory disease of pancreas caused by the release of digestive enzymes into pancreatic interstitium and systemic circulation, and by the production and release of various inflammatory cytokines [1]. The annual incidence of AP ranges from 13 to 45 per 100,000 people [2, 3]. Approximately 20% of patients with AP develop severe acute pancreatitis (SAP), which could lead to a systemic inflammatory response syndrome (SIRS) and multiple organ dysfunction and failure, and has a mortality rate of 8% up to 39% [4–8]. The cause of death is closely related to single or multiple organ complications like acute lung injury (ALI), intestine barrier functional disturbance (IBFD), acute kidney failure (AKI) and so on. Especially pulmonary and intestinal complications are very important death factor in the early stage of SAP [9, 10]. One of the most critical and key pathophysiology changes after SAP was water metabolic abnormalities with pulmonary and intestinal injury. In spite of the recognized important role of water metabolic abnormalities, there is no specific drug to treat edema of lung and intestine.

Aquaporins (AQPs) are a class of membrane water channels, contain six membrane-spanning helical segments and two shorter helical segments that do not span the entire membrane, whose primary function is to facilitate the passive transport of water in various eukaryotes and prokaryotes, and widespread in distribution in organs of mammals [11–13]. To date, there are 13 AQPs and at least 8 of these have been shown to transport water in humans and rodents. The first member of this family, AQP1 (originally known as CHIP28), was identified in erythrocytes in 1991 [14]. Here are some unravel keys link between AQPs and lung and intestine disease. Towne JE demonstrated that the expression levels of AQP-1 and AQP-5 decreased in lungs with pulmonary edema (PE) following viral infection [15]. PE usually occurs in the Traumatic Brain Injury (TBI)-induced ALI patients, and aquaporins (AQPs), particularly AQP1 and AQP4, maintain water balances between the epithelial and microvascular domains of the lung [16]. Sakai et al. study reported that the genes of AQP4 and AQP8 were mainly expressed in the murine colon [17]. It is widely thought that AQPs are involved in diseases that are characterized by alterations in water transport. The regulation of transepithelial fluid transport in the gastrointestinal tract is based on ion transport and water transport by AQPs [18]. AQPs play a crucial role in maintaining water homeostasis of lung and intestine, but specific treatment is quite scare. Thus, effective treatments need to be developed.

Recently, the adjuvant use of herbal medicine ameliorated lung and intestinal injury and led to a marked reduction in morbidity and mortality in China [19, 20]. Dai-Huang-Fu-Zi-Tang (DHFZT), a prescription in traditional Chinese medicine (TCM), composed of three herbs including Radix et Rhizoma Rhei (DH), Radix Aconiti Lateralis Praeparata (FZ) and Radix et Rhizoma Asari (XX), was originally described in the Synopsis of Golden Chamber (Jin Kui Yao Lue), a treatise on febrile and miscellaneous diseases written by the outstanding physician Zhong-Jing Zhang in Han Dynasty. DHFZT has been used to cure AP, acute intestinal obstruction, shock [21, 22]. Recent studies have shown that DHFZT could promote gastrointestinal motility, reduce lung and liver injury, and inhibit cytokine activity and inflammatory responses in SAP [23, 24]. Xiao Liu et al. found that talatisamine, rhein glucoside, rhein isomer methylation, hypaconine, hydroxyl-chrysophanol, emodin glucuronide conjugation, and chrysophanol glucuronide conjugation were the main anti-acute pancreatitis components in DHFZT by UHPLC-ESI-Q-TOF-MS technique [25]. Our previous study also show that DHFZT could enhance the intestinal peristalsis, protect the gastrointestinal barrier function, reduce the bacteria and endotoxin translocation and improve the prognosis of patients with SAP. However, more detailed insights into the molecular mechanism of DHFZT relieving pulmonary and intestinal injury are still not clear after SAP.

So, the study aimed to explore the effect of DHFZT on SAP associated with the pulmonary and intestinal injury. Moreover, to clarify the mechanism involved in AQPs, inflammatory mediators, endotoxin and tissue edema of changes in SAP rat model treated by DHFZT were investigated.

## Methods

### Animals

A total of 64 Sprague - Dawley male rats (age, 5–7 weeks; weight, 250–300 g) were purchased from the Experimental Animal Center of Dalian Medical University. Cages were individually ventilated at 20 ± 2°C and 45–65% relative humidity with a circadian rhythm of 12/12 h. All rats were adaptively fed for 1 weeks before the experiment. All procedures involving animals were conducted in conformity with the National Institute of Health Guide for the Care and Use of Laboratory Animals and were approved by the Animal Research Ethics Committee of affiliated Zhongshan Hospital of Dalian University (2012–60). Anesthetic drugs and all other necessary measures were used to reduce animal suffering during experimental procedures.

### Preparation and quality control of DHFZT

DHFZT is composed of 3 species of herbal plants, each dried crude drug of which were purchased from Tong Ren Tang Group Co., Ltd. (Beijing, China). The formula of DHFZT is described in Table 1, and voucher specimen of *Rheum palmatum* Linn (No.00000022), Aconitum carmichaeli Debeaux (No.01814237), and Asarum heterotropoides F.S chmidt var. mandshuricum (No.00916696) are kept in Institute of Botany, the Chinese Academy of Sciences. To keep the consistency of the herbal chemical ingredients, all of the herbal components were originally obtained from the standard native sources as stated above with GAP grade and the drugs were extracted with standard methods according to Chinese Pharmacopeoia III (edition 2010). According to the original prescription from the〝Jin Kui Yao Lue〞, DH, FZ and XX were mixed in the ration of 3:3:1 (*w*/w). First, FZ were soaked in water (1:25) for 30 min, followed by extraction in boiling water (100 °C) for 1 h. Then DH was added and boiled for 10 min. Finally, XX was added and boiled for 5 min. The DHFZT was concentrated by rotary evaporator (Heidolph Instruments, Germany) and lyophilized to obtain dry extract through freeze-drying system (Labconco, United States) at −80 °C, yielding final 3.72 g (extraction ratio 17.71%), and stored at 4 °C for use. The lyophilized DHFZT extract was dissolved in an appropriate volume of distilled water prior to administrating to rats. In addition, part DHFZT solutions as sample were constant volume by methanol producing a concentration of 5.0 mg/ml and stored at 4 °C for TOF LC/MS analysis.

**Table 1:** Herbal Compositions of DHFZT

Scientific name	Herbal name	Quantity (dry, g)
*Rheum palmatum* Linn	Radix et Rhizoma Rhei (DH)	9.0
Aconitum carmichaeli	Debeaux Radix Aconiti Lateralis Praeparata (FZ)	9.0
Asarum heterotropoides F. Schmidt var. mandshuricum	Radix et Rhizoma Asari (XX)	3.0
Total		21.0

*DHFZT* Dai-Huang-Fu-Zi-Tang, *DH* Dai Huang, *FZ* Fu Zi, *XX* Xi Xin

### Qualitative screening of chemical compositions of DHFZT using accurate-mass time-of-flight liquid chromatography-mass spectrometry (TOF LC/MS)

#### Chromatographic conditions

Sample analyses were carried out on a 6224 TOF LC/MS system (Agilent, CA, USA). Chromatographic separation was carried out on a column of ZORBAX SB-C18 Rapid Resolution HD (3.0 × 100 mm,1.8-Micron) (Agilent). The mobile phase was delivered at a flow rate of 0.20 mL/min consisting of 0.1% formic acid in water (A) and acetonitrile (B) using a gradient program as follows: 3% ~ 20%B (0-15 min), 20% ~ 35%B (15 ~ 25 min), 35% ~ 50%B (25 ~ 30 min), 50% ~ 55% B (30 ~ 35 min),55% ~ 65% (35 ~ 40 min),65% ~ 70% B (40 ~ 45 min),70% ~ 100% B (45 ~ 50 min). The column oven temperature was set at 30 °C throughout the whole analytical procedure. The photodiodearray detector (DAD) was set at 254 nm.

#### Mass spectrometer conditions

The TOF-MS (Agilent 6224 series ion trap mass spectrometer) operation parameters were set as follows: negative ion and positive ion electrospray, nebulizing gas (N_2_) pressure 40psig (1 psi = 6894.8 Pa), drying gas (N_2_) flow rate 9 L/min and temperature 350 °C, applied probe voltage 4000 V. Mass spectrometry was conducted in the full scan and automatic multiple stage fragmentation scan modes over an m/z range of 100 ~ 1500 for MS. Reference ions′ M/z are 122.9856 and 1033.9881. All ions produced were finally introduced into the TOF instrument for accurate mass determination. The data recorded were processed by the Agilent MassHunter Qualitative Analysis B.05.00.

#### Peak selections and data processing

It has been found that the peaks with intensity below 100,000 gave few fragments in the preliminary study; therefore, only the peaks detected with intensity over 100,000 were selected for identifications. The chemical formulas for all parent and fragment ions of the selected peaks were calculated from the accurate mass using a formula predictor by setting the parameters as follows: C [0–60], H [0–120], O [0–30], N [0–10], double bond equivalent (DBE) [0–20], and H/C ratio [0–3]. Other elements such as P, S, and Cl were not considered since they are rarely present in herbal components. All relevant data including peak number, retention time, accurate mass, the predicted chemical formula, Theoretical or Experimental m/z value and corresponding mass error were recorded into an Excel file. The maximum tolerance of mass error was set at 5 ppm when searching for common ions.

### Dose dependant experiment of DHFZT

Forty of Sprague-Dawley rats were used for dose dependant experiment of DHFZT. The lyophilized DHFZT extract was dissolved in distilled water for DHFZT solution (concentration: 100 mg/ml). A dose of 1.0 ml of distilled water was set for the control group (*n* = 8). SAP model rats (*n* = 8) were established by retrograde injections of 5% sodium taurocholate solutions into rat pancreaticobiliary ducts. DHFZT solution were set at 31 mg/kg (low-dose group, *n* = 8), 62 mg/kg (medium-dose group, *n* = 8) and 124 mg/kg (high-dose group, *n* = 8) for the test groups. DHFZT solution was administered through gavage 2 times a day for 2 days. Serum amylase, C-reactive protein, mortalities of all rats were observed.

### Experiments process

**Fig. 1:**
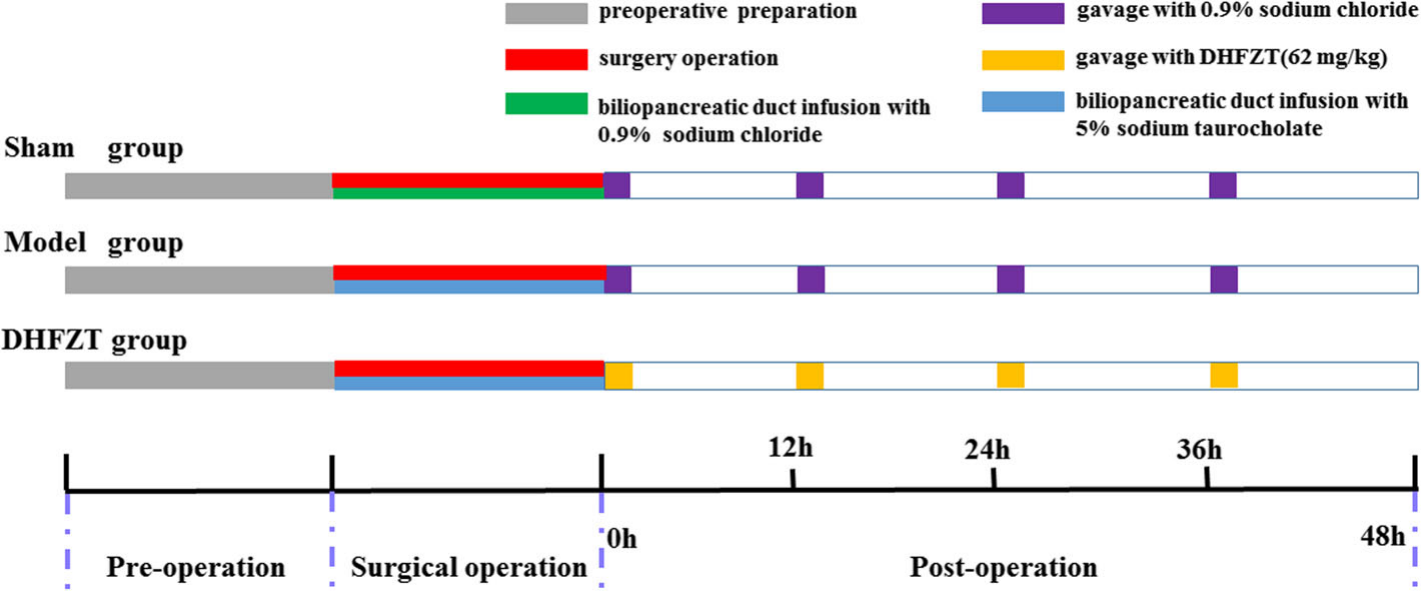
Experimental protocols. All Rats were subjected to surgery operation in sham group, model group and DHFZT group. 5% sodium taurocholate (0.1 ml/100 g) was injected retrogradely into biliopancreatic duct to induce SAP model in rats. 62 mg/kg of DHFZT extract dissolved in 2 ml of 0.9% normal saline was administered to DHFZT group rat at 0, 12, 24, 36 h post-operation. DHFZT: Dai-Huang-Fu-Zi-Tang

The experiment aimed to test whether DHFZT could alleviate pulmonary and intestinal injury via AQP1, AQP5, AQP8 and AQP9. After 1 week of acclimation, a total of 24 rats were randomly divided into three groups, which included sham group (*n* = 8), model group (*n* = 8), DHFZT groups (*n* = 8). The rats were fasted for 8 h, with water deprivation for 4 h before operation. Sodium taurocholate could cause damage of pancreatic acinar cells, which leads to severe acute pancreatitis. Biliopancreatic duct underwent retrograde infusion with 5% sodium taurocholate to induce SAP rat model [26]. Subsequently, the rats were anesthetized by intraperitoneal injection with 10% chloral hydrate (0.3 ml/100 g). An incision was made into the ventral midline for entry into the abdominal cavity, in order to expose biliopancreatic duct. The biliopancreatic duct was occluded at the distal duodenum using a vascular clip. For the model group and DHFZT group, the canal was infused slowly with 5% sodium taurocholate (0.1 ml/100 g) using a 1-ml syringe. DHFZT (62 mg/kg) was administered through gavage at 0, 12, 24 and 36 h following the induction of SAP in the DHFZT group. The rats were abstained from food except water for 12 h post-operation. Blood samples were drawn from the caudal artery for measuring serum amylase, endotoxin, lipase, TNF-α, IL-6 and IL-10 concentrations at 0, 12, 24 and 48 h post-operation. Rats were sacrificed at 48 h, and the lung and ileum tissue were harvested to determine W/D weight ratio and protein expressions of AQP1, 5, 8, and 9 with immunohistochemistry, western blot and immunofluorescence respectively. Right lobe was used for calculating W/D weight ratio, and left lower lobe was used for immunohistochemistry, western blot and immunofluorescence analysis. HE staining was used for assessment of pathologic changes of lung and intestine. The whole experiment protocol showed in Fig. 1.

### Measurement of serum amylase, lipase, endotoxin and cytokines

Blood samples were centrifuged at 15,000 rpm under 4 °C and then stored at −80 °C. Serum amylase and lipase were measured with a TBA-2000FR System (Toshiba, Tokyo, Japan). The serum concentration of endotoxin was measured by EKT-5 M set dynamic Gram-negative bacteria test kit (Jin Shanchuan technology development co., LTD, Beijing, China) through kinetic turbidimetric assay. The serum cytokines (TNF-α, IL-6 and IL-10) were detected using a commercial enzyme-linked immunoabsorbent assay kits (Boster Biological Technology co.ltd, Wuhan, China) according to the manufacturers’ protocols.

### Pulmonary and intestinal W/D ratio

To evaluate the severity of tissue edema, the pulmonary and intestinal W/D ratio were calculated. After euthanasia of rats, the inferior lobe of right lung and a 10 cm of intestinal segment were excised and measured immediately to obtain the “wet weight value”, and then dried in an oven to a constant temperature at 50 °C for 72 h and weighed again to obtain the “dry weight value”. Subsequently, the wet/dry weight ratio was calculated.

### AQPs localization analysis of lung and intestine with immunohistochemistry staining

The imunohistochemistry was carried out to elucidate the tissue localization of AQPs in the lung and intestine. All primary antibodies are anti-rat monoclonal antibodes, and different dilution ratio (AQP1 1:200, AQP5 1:100, AQP8 1:200, and AQP9 1:100). The primary antibodies and goat- anti -rat mouse IgG were purchased from Hebei Bio-high Technology Deve CO., LTD, China. Immunohistochemical staining was performed using the DAB chromogenic detection kit (Hebei Bio-high Technology Deve CO., LTD, China). After washed with PBS, the expression of protein was determined according to the positive chromogenic markers, brown indicated positive expression. All specimens were observed with a microscope (Olympus Corp, Japan) and recorded under the identical optical conditions using ISCapture imaging software after scanning.

### Immunofluorescence analyses of lung and intestine samples

Immunofluorescence staining using specific antibodies was used to quantify the deposition of AQP1, 5, 8 and 9(anti-rat monoclonal antibody). Tissue samples from the right superior lung and intestine were taken, washed in PBS. The tissue were sliced (0.5 cm × 0.5 cm × 1.0 cm) and the slices were immersed in 4% paraformaldehyde (0. 1MPBS, PH7.0 ~ 7.6 and 0.1%DEPC) overnight. The slices were then placed in ethanol and processed immediately. Paraffin-embedded tissue sections (5 μm) were collected on the slide of poly lysine overnight at 60 °C. Plus slides (VWR, Inc.) and the paraffin were removed by dimethylbenzene and ethanol from the sections before performing for immunofluorescence microscopy. The deparaffinized tissue sections were done antigen retrieval solution by water-bath heating for 20 min at 90 °C. The tissue sections were cooled for 20 min at room temperature. The pulmonary and intestinal biopsy sections were incubated overnight with primary antibodies including anti-AQP1 (1:300), anti-AQP5(1:200), anti-AQP8 (1:100), and anti-AQP9 (1:100). These anti-rat monoclonal antibodies were bought from Hebei Bio-high Technology Deve CO., LTD, China. After washing with PBS, the biopsy sections were incubated with goat anti-mouse secondary antibodies (Hebei Bio-high Technology Deve CO., LTD, China) diluted in 2% BSA 1: 1000 in PBS for 1 h. After washing, the gut sections were mounted on glass and photographed in a Zeiss LSM510 confocal microscope (Oberkochen, Germany). Endogenous peroxidase activity was quenched by incubating the tissue sections in 3% hydrogen peroxide for 20 min at room temperature. The tissue sections were blocked with 10% normal goat serum. After the tissue sections were treated with the primary and secondary antibodies (IgG), the tissue sections were coverslipped by neutral resin for fluorescence (IX71 fluorescent inverted microscope, Olympus Corp., Japan).

### Western blot analysis of AQPs

Pulmonary and intestinal tissue were lysed on ice and the cellular plasma proteins were extracted with a protein extraction kit (Pierce Biotechnology, Inc., Rockford, IL, USA), according to the manufacturer’s instructions. Protein concentrations were determined by a Coomassie Brilliant Blue dye-binding assay. Samples were analyzed by SDS-PAGE and electrotransferred to a polyvinylidene diflouride (PVDF) membrane (Millipore Corporation, Billerica, MA, USA). The membrane was blocked for 1 h at room temperature with 5% skimmed milk in Tris-buffered saline with Tween-20 [TBST; 50 mM Tris-HCl (pH 7.4), 150 mM NaCl and 0.1% Tween-20]. The membrane was incubated overnight at 4 °C with anti-rat monoclonal antibody against AQP-1, 5, 8 and 9 respectively in TBST. Washed with TBST, the PVDF membrane was incubated with a biotin-conjugated anti-rabbit antibody (GE Healthcare, Tokyo, Japan), and diluted to 1:3000 in TBST at room temperature for 1 h. Bands of interest were visualized using ECL reagents (PerkinElmer, Waltham, MA, USA) and quantified using the UVP BioImaging system (Biospectrum AC Imaging System, CA, USA) and ImageJ software (National Institutes of Health, USA). β-actin was served as an internal control.

### Histopathological assessment of lung and intestine by HE staining

The left lung and small intestine tissue were fixed with 4% paraformaldehyde overnight, dehydrated in ascending grades of alcohols, embedded in paraffin, and sliced into 5 μm sections. Pulmonary and intestinal tissues were processed with hematoxylin and eosin staining (HE staining). The pathologic changes of pulmonary and intestinal tissue were observed under a light microscope (BX51T-PHD-J11,0 lympus, Tokyo, Japan). Semiquantitative analysis of pulmonary histopathology was performed by scoring the tissues based on pulmonary edema (PE), infiltration of inflammatory cells, alveolar hemorrhage, hyaline membrane, and atelectasis: no lesion, 0; injured area ≤ 25%, 1; injured area 26–50%, 2; injured area 51–70%, 3; injured area 71–90%, 4; injured area > 90%, 5. A total of three fields were randomly selected for each slide and the average was used as the histopathology score. The improved Chiu score method [27] was used for evaluating intestinal injury, the higher scores are, the more severe damage. Criteria of Chiu grading system consist of 5 subdivisions according to the changes of villus and gland of intestinal mucosa: grade 0, normal mucosa; grade 1, development of subepithelial Gruenhagen’s space at the tip of villus; grade 2, extension of the space with moderate epithelial lifting; grade 3, massive epithelial lifting with a few denuded villi; grade 4, denuded villi with exposed capillaries; and grade 5, disintegration of the lamina propria, ulceration, and hemorrhage.

### Statistical analysis

Data are presented as mean ± SEM. Statistical comparison among multiple groups was performed by analysis of variance (ANOVA) followed by Tukey’s Multiple Comparison Test and Bonferroni post tests using the GraphPad Prism 5.0 software (GraphPad Software Inc., San Diego, CA, USA). A value of *P* < 0.05 was considered statistically significant.

## Results

### Information on bioactive compounds of DHFZT

**Fig. 2:**
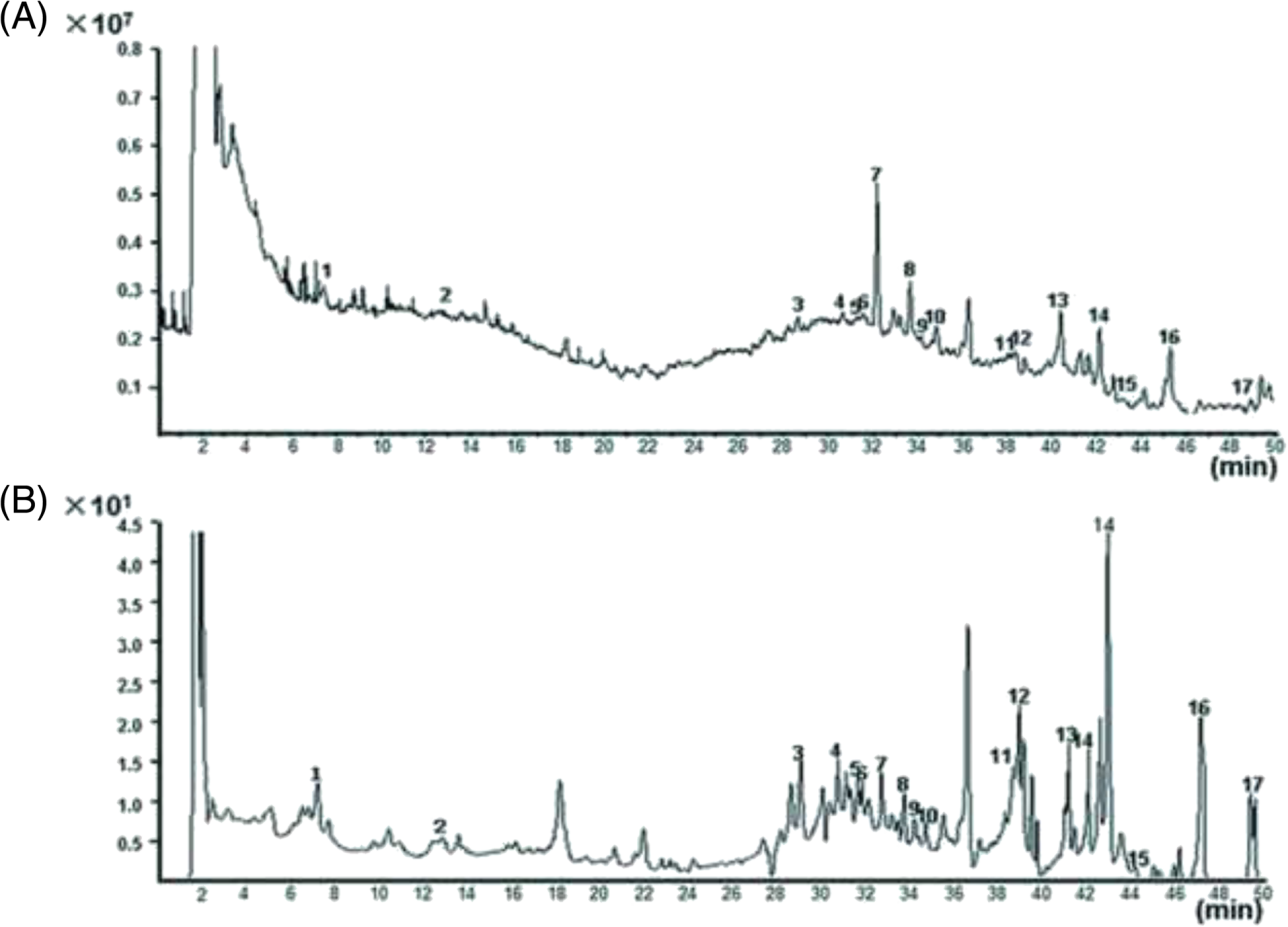
TIC chromatogram and VU chromatogram of the extract of DHFZT in negative﻿ ion mode.﻿﻿﻿﻿﻿﻿ (**a**) total ion current chromatogram, X-axis represents the retention time﻿ (min), Y-axis represents﻿﻿﻿﻿ ion intensity; (**b**) ultraviolet chromatogram﻿, X-axis represents the retention time, Y-axis represents mAU. *TIC* total ion current, *VU* ultraviolet﻿﻿﻿﻿﻿﻿

**Fig. 3:**
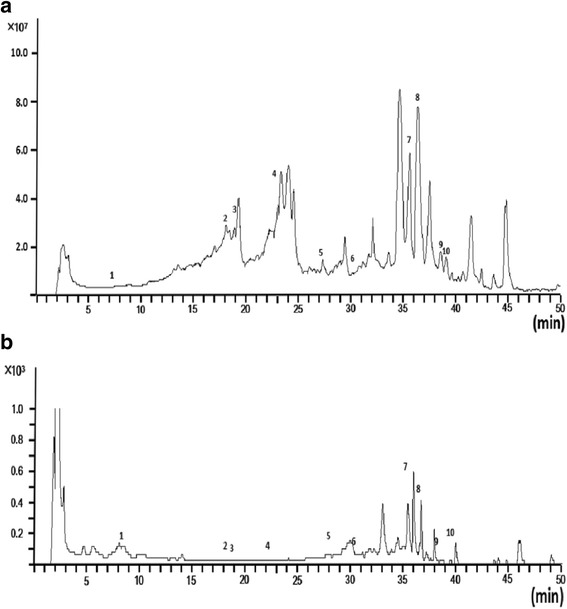
TIC chromatogram and VU chromatogram of the extract of DHFZT in positive ion mode﻿﻿. ﻿(**a**) total ion current chromatogram, X-axis represents the retention time ﻿(min),Y-axis represents﻿﻿﻿﻿ ion intensity;﻿﻿﻿ (**b**) ultraviolet chromatogram, ﻿X-axis represents the retention time﻿ (min), Y-axis represents mAU﻿

TIC chromatogram and VU chromatogram of the extract of DHFZT in negative ion mode and positive ion mode were shown in Figs. 2 and 3. A total of 27 kinds of chemical composition were identified, among which 10 kinds in positive ion mode, and 17 kinds in negative ion mode. In positive ion mode, alkaloid including five kinds of C-19 diterpenoid alkaloids (aconitine, mesaconitine, karakoline, benzoylmes-conitine, benzoylhypaconitine) and 2 kinds C-20 diterpenoid alkaloids (songorine and songoramine) are main constituents of DHFZT. In negative mode, anthraquinones constituents such as Gallic acid, Rhein, emodin and ethers compounds (safrole, α-Asarone and methyleugenol) are main ingredients. The results were shown in Tables 2 and 3.

**Table 2:** Detection of Dai-Huang-Fu-Zi-Tang in positive ion mode

Peak. No	*t*_R_ (min)	Identity	Formula	Selected ion	*m/z*		Error (ppm)
					Theoretical	Experimental	
1	7.275	salsolinol	C_10_H_13_NO_2_	[*M* + H]^+^	180.1019	180.1027	2.02
2	18.136	songorine	C_22_H_31_NO_3_	[*M* + H]^+^	358.2377	358.2383	1.87
3	18.653	karakoline	C_22_H_35_NO_4_	[*M* + H]^+^	378.2639	378.2642	1.66
4	22.173	fuziline	C_24_H_39_NO_7_	[*M* + H]^+^	454.2799	454.2798	−0.19
5	27.312	myristicin	C_11_H_12_O_3_	[*M* + H]^+^	193.0859	193.0857	−0.41
6	30.748	songoramine	C_22_H_29_NO_3_	[*M* + H]^+^	356.2220	356.2221	0.08
7	35.203	benzoylmesaconitine	C_32_H_45_NO_10_	[*M* + H]^+^	604.3116	604.3119	−0.85
8	36.871	aconitine	C_34_H_47_NO_11_	[*M* + H]^+^	646.3222	646.3224	−1.03
9	38.022	mesaconitine	C_33_H_45_NO_11_	[*M* + H]^+^	632.3065	632.3065	−0.61
10	39.741	benzoylhypaconitine	C_32_H_43_NO_10_	[*M* + H]^+^	602.2960	602.2950	1.23

**Table 3:** Detection of Dai-Huang-Fu-Zi-Tang in negative ion mode

Peak.No	*t*_R_ (min)	Identity	Formula	Selected ion	*m/z*		Error (ppm)
					Theoretical	Experimental	
1	7.735	Gallic acid	C_7_H_6_O_5_	[*M* − H]^−^	169.0142	169.0144	−1.13
2	13.308	Hypaconine	C_24_H_39_NO_8_	[*M* − H]^−^	468.2603	468.2598	−0.61
3	29.390	Kakuol	C_10_H1_0_O_4_	[*M* − H]^−^	193.0506	193.0503	1.66
4	31.643	Rhein-8-O-β-D-glucopyranoside	C_21_H_18_O_11_	[*M* − H]^−^	445.0776	445.0778	−0.84
5	31.809	Safrole	C_10_H_10_O_2_	[*M* − H]^−^	161.0608	161.0614	−0.01
6	32.076	Benzoylmesaconine	C_31_H_43_NO_10_	[*M* − H]^−^	588.2824	588.2825	−0.85
7	32.744	Kaempferol 3-glucoside	C_21_H_20_O_11_	[*M* − H]^−^	447.0933	447.0938	−0.95
8	34.012	Sesamin	C_20_H_18_O_6_	[*M* − H]^−^	353.1031	353.1038	1.6
9	35.180	α-Asarone	C_12_H_16_O_3_	[*M* − H]^−^	207.1027	207.1026	0.08
10	35.396	Benzoylaconitine	C_32_H_45_NO_10_	[*M* − H]^−^	602.2971	602.2973	−0.8
11	38.783	Ehrysophanol-8-O-β-D-glueopyranoside	C_21_H_20_O_9_	[*M* − H]^−^	415.1035	415.1038	−0.67
12	38.950	Aloeemodin-ω-β-D-glucopyranoside	C_21_H_20_O_10_	[*M* − H]^−^	431.0984	431.0985	−0.38
13	40.985	Kaempferol	C_15_H_10_O_6_	[*M* − H]^−^	285.0405	285.0409	−1.49
14	42.737	Rhein	C_15_H_8_O_6_	[*M* − H]^−^	283.0248	283.0252	−1.87
15	43.588	Methyleugenol	C_11_H_14_O_2_	[*M* − H]^−^	177.0921	177.0918	4.28
16	45.907	Emodin	C_15_H_10_O_5_	[*M* − H]^−^	269.0455	269.0459	−1.25
17	49.060	Chrysophanic acid	C_15_H_10_O_4_	[*M* − H]^−^	253.0506	253.0501	0.06

### Result of dose dependant experiments of DHFZT

Serum amylase, C-reactive protein and mortality were used for observation of DHFZT dose dependent experiment. During the observation period, Serum amylase and C-reactive protein in medium-dose group were lower than those of low-dose group and high-dose group. In addition, medium-dose DHFZT could decrese the motrality of rats compared to low-dose group and high-dose group. Result of dose dependant experiments of DHFZT showed that medium-dose (62 mg/kg) was appropriate therapeutic dose for subsequent experimental process. The results showed in Table 4.

**Table 4:** Change of Serum amylase, C-reactive protein and mortality at different dose group of DHFZT

Index		Control group (*n* = 8)	SAP group (*n* = 8)	Low-dose group (31 mg/kg, *n* = 8)	Medium-dose group (62 mg/kg, *n* = 8)	High-dose group (124 mg/kg, *n* = 8)
Serum amylase (U/L)		1048 ± 44.43	3338 ± 187.6^a^	3087 ± 116.9^a^	2280 ± 168.6^abc^	3178 ± 124.7^af^
C-reactive protein (ng/ml)		17.86 ± 1.11	55.68 ± 2.55^a^	17.30 ± 1.28^b^	39.81 ± 2.71^bd^	47.74 ± 2.58
Mortality (%)		0.0	37.50	25.00	12.5^c^	25^e^

Versus control group, ^a^ *P* < 0.001; Versus SAP group, ^b^ *P* < 0.001; Versus low-dose group, ^c^ *P* < 0.01, ^d^ *P* < 0.001; Versus medium-dose group, ^e^ *P* < 0.05, ^f^ *P* < 0.001

### DHFZT reduced the level of serum amylase, lipase and endotoxin after SAP

To identify a SAP rat model was established successfully, serum amylase and lipase were determined by ELISA. The serum amylase and lipase in model group were significantly higher than those of sham group at 12, 24 and 48 h (*P*<0.001). After administration of DHFZT, the serum amylase and lipase were reduced in different degree (*P*<0.05 or *P*<0.001) at 12, 24 and 48 h after SAP. In addition, the serum endotoxin was analysed for assessing severity of intestine injury. The presence of endotoxin in the blood would significantly exacerbate systemic inflammation in the SAP animals. The serum endotoxin levels in SAP model group were increased at 24 and 48 h in 5% sodium taurocholate-treated rats in comparison with the level of sham group (*P*<0.001). By contrast, the levels of serum endotoxin in DHFZT group had a very significant reduction at 24, 48 h in DHFZT group compared to model group (both *P* < 0.001). The results were showed in Table 5.

**Table 5.** DHFZT reduced the levels of serum amylase, lipase and endotoxin after SAP in rats

	Groups	0 h	12 h	24 h	48 h
Serum amylase (U/L)	Sham	1146.49 ± 56.16	1087.95 ± 36.74	1095.03 ± 63.42	1155.41 ± 41.51
	Model	1125.95 ± 48.69	2337.60 ± 75.113^a^	3777.14 ± 68.42^a^	3452.73 ± 146.13^a^
	DHFZT	1186.06 ± 49.84	1929.61 ± 90.90^c^	1969.47 ± 87.85^b^	2353.09 ± 103.10^b^
Serum lipase (U/L)	Sham	549.52 ± 20.15	566.48 ± 19.77	581.18 ± 25.85	559.62 ± 20.70
	Model	532.45 ± 36.43	2556.80 ± 191.80^a^	2602.21 ± 185.79^a^	2845.85 ± 114.03^a^
	DHFZT	505.68 ± 52.58	1470.75 ± 82.76^b^	1605.25 ± 66.33^b^	1648.74 ± 70.22^b^
Serum endotoxin (pg/mL)	Sham	12.84 ± 0.55	12.81 ± 0.81	12.60 ± 0.44	13.04 ± 0.96
	Model	12.02 ± 0.78	15.87 ± 0.56	28.14 ± 0.77^a^	52.25 ± 1.82^a^
	DHFZT	12.12 ± 0.59	14.19 ± 0.87	18.13 ± 0.96^b^	30.43 ± 1.46^b^

Versus sham group, ^a^ *P* < 0.001; Versus Model group, ^b^ *P* < 0.001, ^c^ *P* < 0.05

### Effect of DHFZT on water metabolism of lung and intestine after SAP

**Fig. 4:**
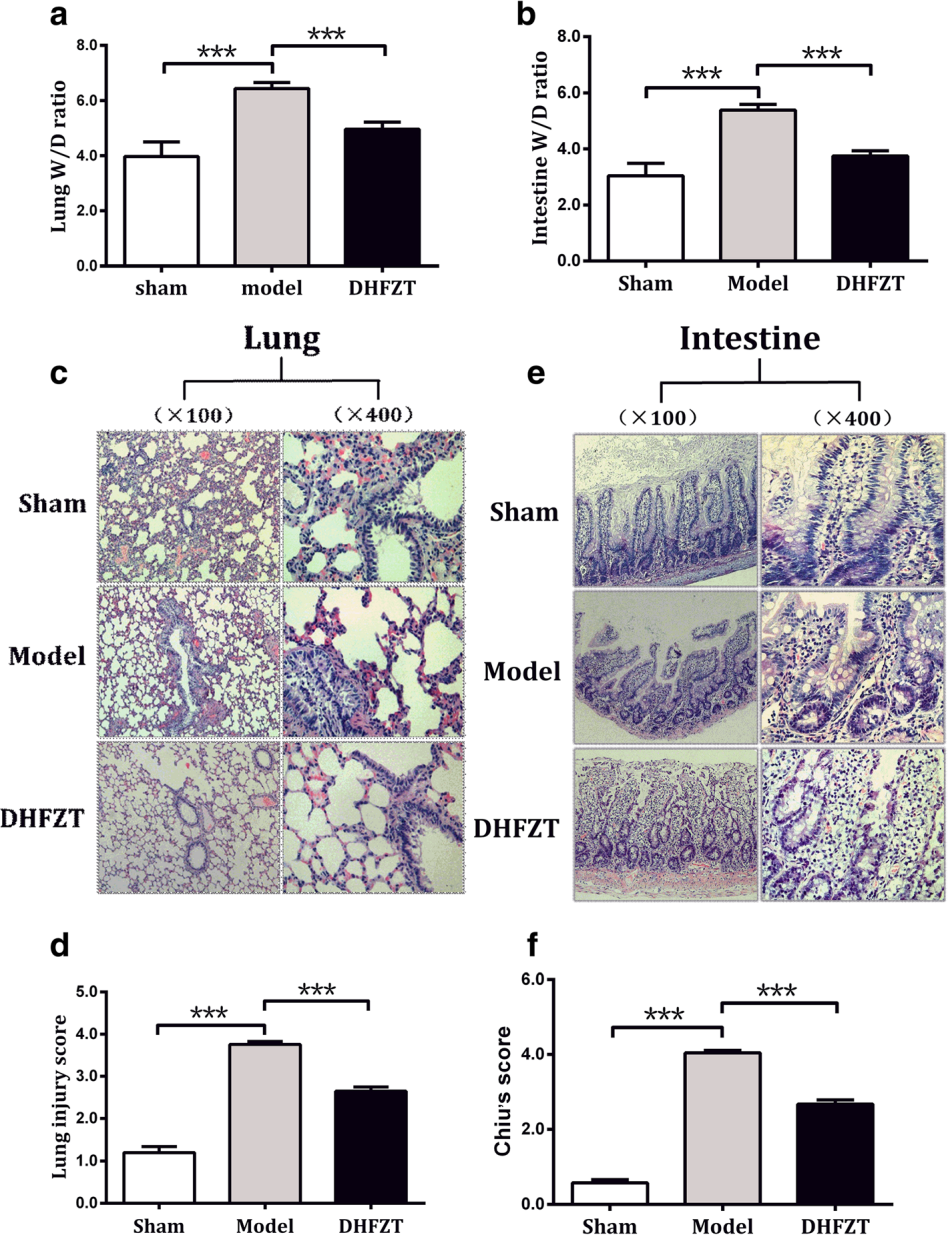
DHFZT attenuates edema and pathological damage of lung and intestine associated with SAP. **a** and **b**:W/D ratio of lung and intestine changes at 48 h in sham group, model group and DHFZT group. **c** and **d**: pathological damage and injury score of lung changes at 48 h in each group. **e** and **f**: pathological damage and injury score of intestine changes at 48 h in each group. Histologic change of lung and intestine were observed under light microscopy (hematoxylin and eosin, ×200 and ×400 respectively). W/D ratio, wet weight value/dry weight value ratio, DHFZT: Dai-Huang-Fu-Zi-Tang group. The results are mean ± SEM. ^***^ *P* < 0.001

Tissue wet/dry (W/D) weight ratio is one of the important indexes to evaluate the water content of the tissue. To detect the effect of DHFZT on water metabolism of lung and intestine, we evaluated the W/D weight ratio. Lung W/D weight ratio was shown in Fig. 4a (F = 30.49, *P* < 0.0001, One-way ANOVA). 5% sodium taurocholate caused a significant increase of lung W/D weight ratio in model group compared with that of sham group (sham group 3.97 ± 0.19 vs model group 6.43 ± 0.22, *P* < 0.001). Whereas the lung W/D weight ratio in DHFZT group was markedly lowered comparison with SAP model group (DHFZT group 4.97 ± 0.26 vs model group 6.43 ± 0.22, *P* < 0.001). W/D weight ratio of intestine was also analysed in Fig. 4b (F = 43.25, *P* < 0.0001, One-way ANOVA). Compared with sham group, the W/D weight ratio of intestine in model group was significantly increased after retrograded injection of 5% odium taurocholate into biliopancreatic duct (sham group 3.04 ± 0.16 vs model group 5.39 ± 0.20, *P* < 0.001). This increase was reversed by administration of DHFZT (model group 5.39 ± 0.20 vs DHFZT group 3.75 ± 0.19, *P* < 0.001). The result showed that DHFZT could evaluate the water content of lung and intestine, and which means a reduction in pulmonary edema.

### DHFZT alleviated histology injury of lung and intestine after SAP

To assess the effect of pathologic histology of DHFZT on 5% sodium taurocholate-induced SAP in rats, the histologic changes in lungs and intestine were examined. Hematoxylin and eosin (HE) staining of the lung tissues showed that tissues from sham group exerted normal structures without histopathologic changes (Fig. 4c). The lung injury score was analysed by one-way ANOVA (F = 277.0, *P* < 0.0001, Fig. 4d). In comparison with sham group, lung tissues from the model group showed a severe pathologic abnormality, including pulmonary interstitial hyperemia, edema and hemorrhage, infiltration of inflammatory cells into alveolar space, and alveolar collapse (lung injury score: sham group 1.20 ± 0.05 vs model group 3.76 ± 0.07, *P <* 0.001, Fig. 4d). However, those pathologic changes were significantly ameliorated by administration of DHFZT (lung injury score: DHFZT group 2.65 ± 0.10 vs model group 3.76 ± 0.07, *P <* 0.001, Fig. 4d). Histopathological assessment and injury severity of small intestines were also performed (Fig. 4e and f). The Chiu′s score was analysed by one-way ANOVA (F = 496.6, *P* < 0.0001, Fig. 4f). Compared with sham group, the rat of model group showed edema in the villi, inflammatory cells infiltration, and damaged areas interspersed with hemorrhage (Chiu′s score: sham group 0.58 ± 0.03 vs model group 4.04 ± 0.07, *P <* 0.001). In addition, the gap between epithelial cells significantly increased and capillaries and lymph vessels were markedly dilated. In comparison with model group, DHFZT significantly attenuated the histological intestine injury (DHFZT group 2.67 ± 0.11 vs model group 4.04 ± 0.07, *P <* 0.001).

### Positioning analysis of AQPs in lung and intestine

**Fig. 5:**
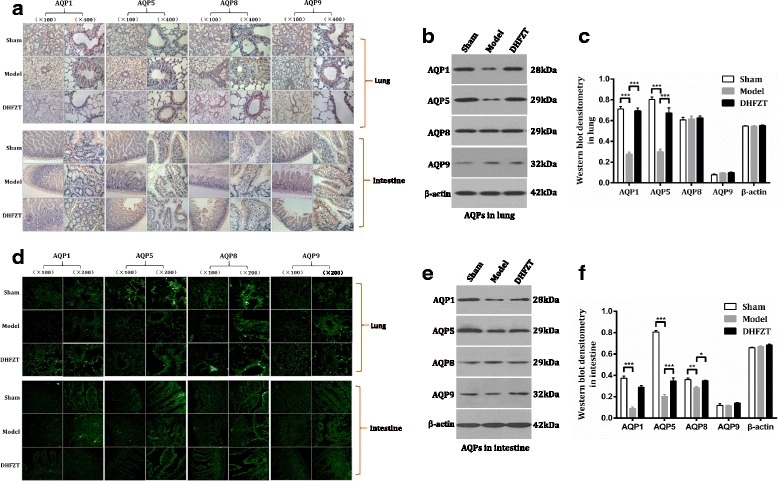
DHFZT regulated the expression of AQPs in lung and intestine after SAP in rats. **a**: distribution and expression of AQPs in lung and intestine at 48 h post-operation by immunohistochemistry (IHC). **b** and **c**: AQPs expression levels in lung by western blot analysis. **d**: expression of AQPs in lung and intestine at 48 h post-operation by immunofluorescence (IF). **e** and **f**: AQPs expression levels in intestine by western blot analysis. ^*^ *P* < 0.05, ^**^ *P* < 0.01, ^***^ *P* < 0.001

As DHFZT treatment improved the edema and injury of lung, we supposed that the activation of AQPs is necessary for water mechanism of lung. To verify our hypothesis and further explore the mechanism of water metabolism of lung, Immunohistochemistry (IHC) and immunofluorescence (IF) were used for positioning analysis ofAQP1, AQP5, AQP8 and AQP9 protein. IHC and IF showed that AQP1 is expressed in all vascular endothelial cells, AQP5 in the alveolar type I cells, AQP8and AQP9 in the bronchial epithelial cells in lung. The fluorescence intensity of AQP9 expression was much less than that of AQP1, AQP5 and AQP8 in lung. In intestine, the distribution of AQP1 appeared to be localized to the submucosa layer of vessel, and AQP8 are expressed in microvillus, AQP5 and AQP9 in the epithelial cells of the intestine. The results were showed in Fig. 5a and d.

### DHFZT regulated the expression of AQPs in lung and intestine

To further observe the effect of DHFZT on AQPs pulmonary and intestinal AQP1, AQP5, AQP8 and AQP9 protein, western blot and immunofluorescence were for estimation of the protein expression levels of all four AQPs in each groups. Western blot showed a dramatic reduction in the levels of pulmonary AQP1, AQP5 (both *P <* 0.001) at 48 h after SAP model. There is little difference in AQP8 and AQP9 between model group and sham group (*P* > 0.05, two-way ANOVA, in Fig. 5c). Compared with model group, DHFZT observably increased the levels of AQP1 (*P* < 0.001), AQP5 (*P* < 0.001), not including AQP8 (*P* > 0.05) and AQP9 (*P* > 0.05) in lung. Immunofluorescence results also demonstrated the change trend. The results were showed in Fig. 5b and c.

Strong expression of intestinal AQP1, AQP5 and AQP8 in sham group was also observed by western blot, and AQP9’s expression was significantly lower than other three AQPs in model group. Expression of intestinal AQP1, AQP5 and AQP8 in model group had an obvious reduce compared to that of sham group (all *P* < 0.01, two-way ANOVA). AQP9 expression in intestine was just the opposite, but there is no obvious difference between sham group and model group. After the treatment with DHFZT, the expression of AQP1, AQP5 and AQP8 in DHFZT group was increased compared to that of model group respectively (*P* < 0.001, *P* < 0.001 and *P* < 0.05). There was no change in AQP9 expression after the treatment of DHFZT. The results were showed in Fig. 5d, e and f.

### Changes of serum level of TNF-α, IL-10, and IL-6

**Fig. 6:**
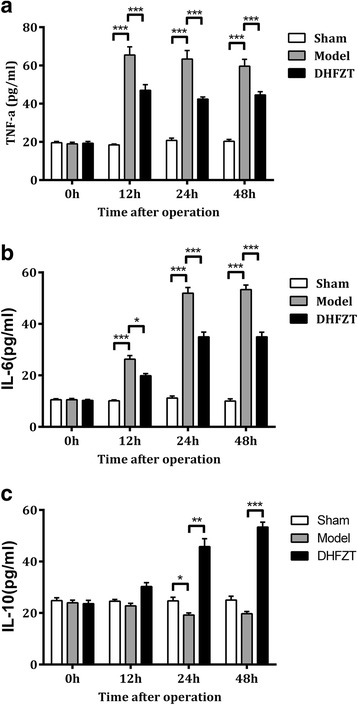
DHFZT affected the changes of serum concentrations of TNF-α, IL-6 and IL-10 after SAP. ﻿Sham: sham group; Model: Severe acute pancreatitis group; DHFZT: DHFZT group.﻿ Serum concentrations of TNF-α, IL-10, and IL-6 were determined using enzyme linked immunosorbent assay. TNF-α: tumor necrosis factor-α; IL-6: interleukin-6; IL-10: interleukin-10. Data are expressed as means ± SEM. ^*^ *P* < 0.05, ^**^ *P* < 0.01, ^***^ *P* < 0.001

The level of serum TNF-α and IL-6 in the model group was significantly higher than that in sham group at 12, 24, 48 h after SAP (all *P* < 0.001). There were no significant statistical changes in spite of the level of IL-10 reduced lightly in the model group compared to that of sham group (*P* > 0.005 except 24 h post-operation 0.05). Treatment with DHFZT significantly reduced TNF-α and IL-6 levels and increased IL-10 levels. DHFZT markedly decreased pro-inflammatory factor release and increased the anti-inflammatory factor. The results showed in Fig. 6 (TNF-α, F = 21.66, *P* < 0.001; IL-6, F = 67.47, *P* < 0.001; IL-10:F = 33.17,*P* < 0.001, two-way ANOVA).

## Discussion

The principal goal of this study was to assess whether DHFZT relieved the pulmonary and intestinal injury by regulating the expression of AQP1, AQP5, AQP8 and AQP9 after SAP. We have identified SAP induced by 5% sodium taurocholate could cause abnormal metabolic disorder of water and injury of lung and intestine, and significantly down-regulated expression of AQP1 and AQP5 in lung, and AQP1, AQP5 and AQP8 in intestine, and increase release of inflammatory factors. Furthermore, the results of immunohistochemistry, immunofluorescence and western bot showed that DHFZT promoted the expression of AQP1, AQP5 or AQP8 in lung and intestine after SAP in rats. DHFZT alleviated W/D weight ratio and histology injury of lung and intestine caused by SAP. In addition, DHFZT also significantly reduced the inflammatory reaction by lowering the expression of inflammatory mediators such as TNF-α, IL-6 and increasing IL-10 level at 24 h, 48 h after SAP. Based on this study and previous work, DHFZT could alleviate edema and injury of lung and intestine induced by SAP via regulating AQP1, AQP5 and/or AQP8 in lung and intestinal tissue.

Acute lung injury (ALI) is a clinical syndrome characterized by highly permeable lung edema. Pulmonary edema is an imbalance of lung tissue fluid formation and reflux. A great deal of tissue fluid could not be absorbed in lung lymph and pulmonary venous system and accumulate in the alveoli and 22 bronchioles, which result in pulmonary ventilation and ventilation dysfunction. So far, despite years of research in ALI, the mechanism involved in the disease pathogenesis of ALI has not been completely elucidated. The pathological characteristics of pulmonary edema include fluid accumulation in the alveolar and interstitial lung. Alveolar water transport was believed to be associated with the active transport of sodium, because of the lack of awareness of the function of aquaporins (AQPs) in lung tissues. AQPs are ubiquitous in nearly all organisms, mediating selective and rapid flux of water across biological membranes. Singha et al. studies have shown that AQP is involved in the pathogenesis of lung edema [28]. Bai et al. also found a 10-fold decrease in the water permeability of the alveolar capillary membrane barrier following an AQP-1 gene knockout [29]. Meanwhile, the water permeability was decreased 14 to 16 times after AQP-1 and AQP-4 knockout in mice. These indicated that AQPs selectively allowed the passage of water molecules and mediated the transmembrane transport of free water molecules. Therefore, these proteins are believed to closely influence the lung water balance and the occurrence of lung edema. The expression of AQP-1 was significantly decreased after subjecting them to 4–12 h of ALI induced by SAP [30]. In this study, western blot revealed that lung tissue mainly expressed the AQP1, AQP5, and AQP5, but AQP9 expressed very little. The immunohistochemistry and immunofluorescence assessed the distribution and intensity of protein expression of these AQPs. AQP-1 was mainly expressed in the endothelial cells of capillary around the bronchi and alveolar type II epithelial cells. Pulmonary AQP5 is expressed in the apical membrane of type I alveolar epithelial cells and in apical membrane of acinar epithelial cells in submucosal glands, and AQP8, 9 in the bronchial epithelial cells.

One of severe complication of SAP is often intestinal barrier damage. A few studies emphasized that the major damage occurring in SAP patients is not necrosis of the pancreas, but intestinal bacterial translocation, enterogenic endotoxemia and secondary pancreatic infection [31, 32]. The small intestine may become injured during SAP due to alterations in microcirculation associated with fluid loss, hypovolemia, splanchnic vasoconstriction and ischemia-reperfusion injury, and failure of the small intestine tends to aggravate the course of SAP [33]. In this study, we found that intestinal W/D weight ratio and pathological score were significantly increased at 48 h after SAP, which indicated that intestinal edema and injury appeared after the onset of pancreatitis. Our results showed that AQP1, AQP5 and AQP8 in model group were down-regulated post-SAP compared with sham group, while AQP9 was no difference between them. Although AQP9 was still expressed in intestinal submucosa cells or glands, expression level of that was far less than other AQPs. Yuan-Hung Wang found that 6 AQP genes (AQP1, 3, 5, 7, 8, and 11) was identified in the rat intestine [34]. AQP1 was mainly expressed in endothelium of capillaries, small vessels, and central lacteal of the intestine by immunohistochemistry analysis [35, 36]. In this study, AQP-1 expression was markedly reduced compared to the sham group at different time points, with the lowest level detected at 48 h after SAP induction.

The AQPs in mammals are divided into three subgroups: AQPs with water-specific channels (AQP0, AQP1, AQP2, AQP4, AQP5, AQP6, AQP8), aquaglyceroporins that conduct small neutral solutes like glycerol and urea in addition to polar water molecules channels (AQP3, AQP7, AQP9), and a new subfamily, the super-aquaporins (AQP11, AQP12). AQPs play a pivotal role in maintaining water homeostasis and glycerol metabolism in the respiratory system and intestinal tract [37–39]. In this study, we chose three water-specific channels (AQP1, AQP5, AQP8) and one water-specific channels (AQP9) as targets of this study, which was for observing whether these AQPs were associated with edema and injury of in lung and intestine, and regulated by DHFZT. The present study revealed that there were different location and expression level of AQP1, AQP5, AQP8 and AQP9 both in lung and intestine. Whether in lung or in intestine, the expression of AQP1, AQP5, AQP8 were significantly higher than AQP9 in sham group. SAP induced aggravated pulmonary and intestinal edema. The protein expression of AQP1 and AQP5 both in lung and intestine, and AQP8 only in intestine were significantly downregulated after SAP, when compared with the sham group in rats. AQPs facilitated the secretion of water into acini of glands following the creation of an osmotic gradient by the active secretion of solutes. In this study, the low expression of AQP1 and AQP5 don’t successfully eliminate alveoli and interstitial water after SAP, therefore, which leads to the pulmonary edema and injury. The lower expression levels of AQP1, AQP5 and AQP8 with the exception of AQP9 in the lung and intestine may be the important pathogenic factors of pulmonary and intestinal edema and injury induced by SAP.

DHFZT is an effective traditional prescription used for the treatment of SAP and its complications, which has advantages of a low cost and a high therapeutic effect [23]. The active components and the spectrum-effect relationships of DHFZT in rat were identified by using UHPLC–ESI–Q–TOF–MS method after oral administration of DHFZT [25]. This study showed that 27 kinds of chemical composition were identified, including 10 kinds in positive ion mode and 17 kinds in negative ion mode. In positive ion mode, aconitine, mesaconitine, karakoline, benzoylmes-conitine, benzoylhypaconitine, songorine and songoramine were main constituents of DHFZT. In negative mode, Gallic acid, Rhein, emodin, safrole, α-Asarone and methyleugenol were main ingredients. Modern clinical and experimental studies showed that the mechanisms of alleviating SAP by DHFZT include improving pulmonary and intestinal function, promoting the excretion of endotoxins and inhibiting the release of inflammatory mediums and cytokines, in order to prevent organ damage. The current study confirmed that DHFZT alleviated the pathological damage of lung and intestine induced by SAP. Furthermore, DHFZT was shown to improve pulmonary and intestinal edema, excessive accumulation of interstitial fluid and overflow of the alveolar lumen fluid, by regulating the protein expression of AQP1 and AQP5, rather than AQP9. In addition, DHFZT could also upregulate the level of AQP8 in intestine for reducing intestinal water metabolic disorder. These data would suggest that administration of DHFZT may reduce the extent of edema and injury of lung and intestine by upregulating the water-specific channels, AQP1, AQP5 in lung and AQP1, AQP5, AQP8 in intestine without aquaglyceroporin channel (AQP9).

Excessive systemic inflammatory response in SAP leads to distant organ damage and multiple organ dysfunction syndromes (MODS), which is the primary cause of morbidity and mortality [40]. Local recruitment and activation of inflammatory cells in injured pancreas may lead to the production of pro-inflammatory cytokines, such as IL-6 and TNF-α, as well as anti-inflammatory IL-4 and IL-10.The imbalance of pro-inflammatory and anti-inflammatory cytokines could lead to the inflammatory cascade and injure intestine and lung tissue [10, 41]. Xue D et al. [42, 43] demonstrated that TNF-α is an important index for the severity of SAP. A study by Gao Z revealed that marked negative linear association was observed between the expression levels of AQP1 and TNF-α in the present study [44]. DHFZT possesses various pharmacological effects, such as anti-inflammatory and anti-oxidative effects [45–48]. As a famous traditional Chinese prescription, it has been widely used to treat various inflammatory diseases, including SAP. The action of DHFZT on SAP could be attributed to its effects on the intestinal peristalsis, the bacteria and endotoxin translocation, and the anti-inflammatory activity. In this study, we examined the value of TNF-α, IL-6, and IL-10 as predictors of inflammation in SAP rats. Our result showed that the level of serum TNF-α and IL-6 in SAP model group was markedly higher than that of sham group at 12, 24, 48 h. DHFZT could increase the expression of anti-inflammatory cytokines (IL-10) and inhibit the expression of pro-inflammatory cytokines (TNF-α and IL-6) to ameliorate the histopathological damage of lung and intestine after SAP. These data support a DHFZT regulated systemic pro-inflammatory media/anti-inflammatory media balance in rats. Similar results can be found in other studies. A study by Lu et al. also demonstrated that DHFZT could effectively reduce serum TNF-α, IL-6 expression and improve the prognosis of patients with SAP in Intensive Care Unit [23]. These protective effects of DHFZT could be closely related to the pharmacological effects of DHFZT and its component herbs. DHFZT is composed of three different medicinal herbs, and the major phytochemical components of DHFZT include emodin, aloe emodin, rhein, physcion, chrysophanol, benzoylmesaconine, benzoylaconine, benzoylhypaconine, gallic acid, and asarinin. In particular, *Rheum officinale* Baill showed protective effects against experimental SAP [49, 50], and emodin is one of the most active ingredients in *Rheum officinale* Baill., which was considered to be a major protective agent in the pancreas and liver [51]. Treatment with DHFZT could significantly reduce the mRNA expression of IL-6 caused by SAP, and attenuated the up-regulated mRNA expression level of TNF-α at the dose of 48 mg/kg [24]. DHFZT inhibited increase in the serum plasma concentrations of TNF-α and IL-1β after SAP and elevated that of IL-10, suggesting that the anti-inflammatory effects of DHFZT might be responsible for the prevention of pulmonary and intestinal complication induced by SAP in rats. Gram-negative infections may be an important predisposing factor for ALI or ARDS in acute pancreatitis [52]. Bacterial endotoxin, as shown by experimental and clinic evidence, is an important trigger factor of SAP resulting in remote organ and multiple organ dysfunction syndrome (MODS). Without a doubt, it is known that pulmonary edema partly is mediated by inflammation. Circulating endotoxin induces the release of cytokines such as TNF-α and IL-1β, which can increase permeability of the endothelium and the alveolar epithelium [53, 54]. We found that serum endotoxin level at 24 h, 48 h in model group was dramatically increased than that of sham group, which was reversed by treatment with DHFZT.

## Conclusions

﻿﻿This is the first study that 27 kinds of chemical composition including 10 kinds in positive ion mode and 17 kinds in negative ion mode were identified by accurate-mass time-of-flight liquid chromatography-mass spectrometry (TOF LC/MS). ﻿﻿﻿W﻿e have identified the distribution feature and change of AQP 1, 5, 8 and 9 in lung and intestine in rats. Subsequently this study show that DHFZT could reduce the pulmonary and intestinal edema and injury induced by SAP through regulating the expression of AQP1, AQP5, AQP8 instead of AQP9. In addition, our experimental data revealed that DHFZT had anti-inflammatory responses in rats with SAP, which further demonstrate the advantage of traditional Chinese medicine decoction. ﻿Even though this study is just an animal experiment,the findings should be appreciated as limited and preliminary.﻿﻿ The study will help us understand the mechanism of DHFZT in the treatment of patients with SAP in clinical practice. Further research could build on the current study.﻿﻿

## Abbreviations

ALI: Acute lung injury
AQP: Aquaporin
DHFZT: Dai-Huang-Fu-Zi-Tang
IL-10: Interleukin 10
IL-6: Interleukin 6
SAP: Severe Acute Pancreatitis
TCM: Traditional Chinese medicine
TNF-α: Tumor necrosis factor –α

## References

[1] Lankisch PG, Apte M, Banks PA (2015). Acute pancreatitis. Lancet (London, England).

[2] Yadav D, Lowenfels AB (2013). The epidemiology of pancreatitis and pancreatic cancer. Gastroenterology.

[3] Andersson R, Andersson B, Haraldsen P, Drewsen G, Eckerwall G (2004). Incidence, management and recurrence rate of acute pancreatitis. Scand J Gastroenterol.

[4] Heinrich S, Schafer M, Rousson V, Clavien PA (2006). Evidence-based treatment of acute pancreatitis: a look at established paradigms. Ann Surg.

[5] Windsor JA, Petrov MS (2013). Acute pancreatitis reclassified. Gut.

[6] Petrov MS, Shanbhag S, Chakraborty M, Phillips AR, Windsor JA (2010). Organ failure and infection of pancreatic necrosis as determinants of mortality in patients with acute pancreatitis. Gastroenterology.

[7] Banks PA, Freeman ML (2006). Practice guidelines in acute pancreatitis. Am J Gastroenterol.

[8] Besselink MG, van Santvoort HC, Boermeester MA, Nieuwenhuijs VB, van Goor H, Dejong CH, Schaapherder AF, Gooszen HG (2009). Timing and impact of infections in acute pancreatitis. Br J Surg.

[9] Andersson B, Olin H, Eckerwall G, Andersson R (2006). Severe acute pancreatitis-outcome following a primarily non-surgical regime. Pancreatology.

[10] Zhou MT, Chen CS, Chen BC, Zhang QY, Andersson R (2010). Acute lung injury and ARDS in acute pancreatitis: mechanisms and potential intervention. World J Gastroenterol.

[11] Agre P, Brown D, Nielsen S (1995). Aquaporin water channels: unanswered questions and unresolved controversies. Curr Opin Cell Biol.

[12] Cheng A, van Hoek AN, Yeager M, Verkman AS, Mitra AK (1997). Three-dimensional organization of a human water channel. Nature.

[13] Sogami M, Era S, Murakami M, Seo Y, Watari H, Uyesaka N (2001). Application of the transition state theory to water transport across cell membranes. Biochim Biophys Acta.

[14] Preston GM, Agre P (1991). Isolation of the cDNA for erythrocyte integral membrane protein of 28 kilodaltons: member of an ancient channel family. Proc Natl Acad Sci U S A.

[15] Towne JE, Harrod KS, Krane CM, Menon AG (2000). Decreased expression of aquaporin (AQP)1 and AQP5 in mouse lung after acute viral infection. Am J Respir Cell Mol Biol.

[16] Chen GS, Huang KF, Huang CC, Wang JY (2015). Thaliporphine derivative improves acute lung injury after traumatic brain injury. Biomed Res Int.

[17] Sakai H, Sagara A, Matsumoto K, Hasegawa S, Sato K, Nishizaki M, Shoji T, Horie S, Nakagawa T, Tokuyama S (2013). 5-fluorouracil induces diarrhea with changes in the expression of inflammatory cytokines and aquaporins in mouse intestines. PLoS One.

[18] Kunzelmann K, Mall M (2002). Electrolyte transport in the mammalian colon: mechanisms and implications for disease. Physiol Rev.

[19] Xia Q, Huang Z, Jiang J, Cheng G, Yang X, Tang W (2006). Yi-Huo-Qing-Xia method as the main therapy in integrated traditional Chinese and western medicine on severe acute pancreatitis: a report of 1161 cases. Chin J Integr Tradit West Med Intensive Crit Care.

[20] Wan MH, Li J, Gong H (2011). Clinical observation on the efect of dexamethasone and Chinese herbal decoction for purgationin severe acute pancreatitis patients. Chin J Integr Med.

[21] Lu X, Kang X, Zhan L, Lv C, Fan Z, Wang Y, Ali R, Lv C, Li S, Mu J (2014). Dai Huang Fu Zi Tang could ameliorate intestinal injury in a rat model of hemorrhagic shock by regulating intestinal blood flow and intestinal expression of p-VASP and ZO-1. BMC Complement Altern Med.

[22] Liang XX, Zhang BG, Liu Q (2008). Pharmacodynamic research and clinical application of Dahuang Fuzi Tang. Chin Trad Pat Med.

[23] Lu XG, Zhan LB, Kang X, Liu L, Li YZ, Yu J, Fan ZW, Bai LZ, Ji CY, Wang XZ (2010). Clinical research of Dahuang Fuzi decoction in auxiliary treatment of severe acute pancreatitis: a multi-center observation in 206 patients. Chin Crit Care Med.

[24] Wu L, Li H, Zheng SZ, Liu X, Cai H, Cai BC (2013). Da-Huang-Fu-Zi-Tang attenuates liver injury in rats with severe acute pancreatitis. J Ethnopharmacol.

[25] Liu X, Wang XL, Wu L, Li H, Qin KM, Cai H, Pei K, Liu T, Cai BC (2014). Investigation on the spectrum-effect relationships of Da-Huang-Fu-Zi-Tang in rats by UHPLC-ESI-Q-TOF-MS method. J Ethnopharmacol.

[26] Aho HJ, Koskensalo SM, Nevalainen TJ (1980). Experimental pancreatitis in the rat. Sodium taurocholate-induced acute haemorrhagic pancreatitis. Scand J Gastroenterol.

[27] Chiu CJ, McArdle AH, Brown R, Scott HJ, Gurd FN (1970). Intestinal mucosal lesion in low-flow states. I. A morphological, hemodynamic, and metabolic reappraisal. Arch Surg.

[28] Singha O, Kengkoom K, Chaimongkolnukul K, Cherdyu S, Pongponratn E, Ketjareon T, Panavechkijkul Y, Ampawong S (2013). Pulmonary edema due to oral gavage in a toxicological study related to aquaporin-1, −4 and −5 expression. J Toxicol Pathol.

[29] Bai C, Fukuda N, Song Y, Ma T, Matthay MA, AS V (1999). Lung fluid transport in aquaporin-1 and aquaporin-4 knockout mice. J Clin Invest.

[30] Liu L, Du L, Chen Y, Qin S, Liang Q, Zou X, Liang X, Jiang J, Chen Q, Wang K (2014). Down-regulation of aquaporin1 (AQP1) by peptidoglycan via p38 MAPK pathways in primary rat pleural mesothelial cells. Exp Lung Res.

[31] Samel S, Lanig S, Lux A, Keese M, Gretz N, Nichterlein T, Sturm J, Lohr M, Post S (2002). The gut origin of bacterial pancreatic infection during acute experimental pancreatitis in rats. Pancreatology.

[32] Juvonen PO, Alhava EM, Takala JA (2000). Gut permeability in patients with acute pancreatitis. Scand J Gastroenterol.

[33] Capurso G, Zerboni G, Signoretti M, Valente R, Stigliano S, Piciucchi M, Delle Fave G (2012). Role of the gut barrier in acute pancreatitis. J Clin Gastroenterol.

[34] Wang YH, Liu TT, Kung WM, Chen CC, Wen YT, Lin IC, Huang CC, Wei L (2015). Expression of aquaporins in intestine after heat stroke. Int J Clin Exp Pathol.

[35] Bao C, Hu S, Zhou G, Tian Y, Wu Y, Z. S (2010). Effect of Carbachol on intestinal mucosal blood flow, activity of Na+−K+−ATPase, expression of Aquaporin-1, and intestinal absorption rate during Enteral resuscitation of burn shock in rats. J Burn Care Res.

[36] Bao C-M, Hu S, Geng S-J, Wu J, Che J-W, Tian Y-J, Sheng Z-Y (2008). Effect of carbachol on expression of TNF-a and aquaporin-1 in small intestine during enteral resuscitation of scald injury in rats. World Chin J Digestol.

[37] Agre P, King LS, Yasui M, Guggino WB, Ottersen OP, Fujiyoshi Y, Engel A, Nielsen S (2002). Aquaporin water channels--from atomic structure to clinical medicine. J Physiol.

[38] Nielsen S, King LS, Christensen BM, Agre P (1997). Aquaporins in complex tissues. II. Subcellular distribution in respiratory and glandular tissues of rat. Am J Phys.

[39] Kreda SM, Gynn MC, Fenstermacher DA, Boucher RC, Gabriel SE (2001). Expression and localization of epithelial aquaporins in the adult human lung. Am J Respir Cell Mol Biol.

[40] Hong W, Dong L, Huang Q, Wu W, Wu J, Wang Y (2011). Prediction of severe acute pancreatitis using classification and regression tree analysis. Dig Dis Sci.

[41] Escobar J, Pereda J, Arduini A, Sandoval J, Sabater L, Aparisi L, Vento M, Lopez-Rodas G, Sastre J (2010). Role of redox signaling, protein phosphatases and histone acetylation in the inflammatory cascade in acute pancreatitis. Therapeutic implications. Inflamm Allergy Drug Targets.

[42] Xue D, Zhang W, Zhang Y, Wang H, Zheng B, Shi X (2006). Adjusting effects of baicalin for nuclear factor-kappaB and tumor necrosis factor-alpha on rats with caerulein-induced acute pancreatitis. Mediat Inflamm.

[43] Li ZL, Wu CT, Lu LR, Zhu XF, Xiong DX (1998). Traditional Chinese medicine Qing Yi Tang alleviates oxygen free radical injury in acute necrotizing pancreatits. World J Gastroenterol.

[44] Gao Z, Xu J, Sun D, Zhang R, Liang R, Wang L, Fan R (2014). Traditional Chinese medicine, Qing Ying Tang, ameliorates the severity of acute lung injury induced by severe acute pancreatitis in rats via the upregulation of aquaporin-1. Exp Ther Med.

[45] Park KH, Park M, Choi SE, Jeong MS, Kwon JH, Oh MH, Choi HK, Seo SJ, Lee MW (2009). The anti-oxidative and anti-inflammatory effects of caffeoyl derivatives from the roots of Aconitum Koreanum R. RAYMOND. Biol Pharm Bull.

[46] Quang TH, Ngan NT, Minh CV, Kiem PV, Tai BH, Thao NP, Song SB, Kim YH (2012). Anti-inflammatory and PPAR transactivational effects of secondary metabolites from the roots of Asarum Sieboldii. Bioorg Med Chem Lett.

[47] Silveira JP, Seito LN, Eberlin S, Dieamant GC, Nogueira C, Pereda MC, Di Stasi LC (2013). Photoprotective and antioxidant effects of Rhubarb: inhibitory action on tyrosinase and tyrosine kinase activities and TNF-alpha, IL-1alpha and alpha-MSH production in human melanocytes. BMC Complement Altern Med.

[48] Wang J, Qiao LF, Yang G (2008). Role of Shenfu injection in rats with systemic inflammatory response syndrome. Chin J Integr Med.

[49] Feng Z, Fei J, Wenjian X, Jiachen J, Beina J, Zhonghua C, Xiangyi Y, Shaoying W (2012). Rhubarb attenuates the severity of acute necrotizing pancreatitis by inhibiting MAPKs in rats. Immunotherapy.

[50] Zheng SH, Tong QY, Zhu ZY, Li ZY, You H (2011). Effect of Rhubarb administered via different routes on blood inflammatory cytokines levels of patients with severe acute pancreatitis. Zhongguo Wei Zhong Bing Ji Jiu Yi Xue.

[51] Muto A, Hori M, Sasaki Y, Saitoh A, Yasuda I, Maekawa T, Uchida T, Asakura K, Nakazato T, Kaneda T (2007). Emodin has a cytotoxic activity against human multiple myeloma as a Janus-activated kinase 2 inhibitor. Mol Cancer Ther.

[52] Gray KD, Simovic MO, Chapman WC, Blackwell TS, Christman JW, May AK, Parman KS, Stain SC (2003). Endotoxin potentiates lung injury in cerulein-induced pancreatitis. Am J Surg.

[53] Xing J, Birukova AA (2010). ANP attenuates inflammatory signaling and rho pathway of lung endothelial permeability induced by LPS and TNFalpha. Microvasc Res.

[54] Ganter MT, Roux J, Miyazawa B, Howard M, Frank JA, Su G, Sheppard D, Violette SM, Weinreb PH, Horan GS (2008). Interleukin-1beta causes acute lung injury via alphavbeta5 and alphavbeta6 integrin-dependent mechanisms. Circ Res.

